# Supplementary material for: Navigating cancer: Insights from patient journey mapping
Source: PLoS One. 2026 Mar 25;21(3):e0327113. doi: 10.1371/journal.pone.0327113 (PMC13016344; doi:10.1371/journal.pone.0327113)
Supplement: Attachment — Submitted filename: NAVIGATING CANCER CARE REVIEWERS REPORT NM.docx [file pone.0327113.s001.docx]

**Navigating Cancer: Insights from Patient Journey Mapping**

Thank you for the opportunity to review this interesting and important topic. Research shows that cancer is the world’s biggest killer, with 10 million deaths per year due to the disease.

**Comments**

**Abstract**

The abstract does provide a background and introduction of the study.

**Main study**

**Introduction**

The presence of important and current background information on the study topic is noted and appreciated.

**Methods**

Mapping patient journey is an interesting and unique research design appropriate to understand patients’ cancer journeys across the continuum, highlight disparities, and gaps in access and care. Sampling, data collection instruments, data collection and analysis methods have been clearly described and applied well to yield the reported results. However, researchers are not explicit as to how participant recruitment and data collection was done in the three different provinces. How it is written can confuse the reader, e. g, “map of the municipality where data collection took place” can make one think data collection took place in one province and one municipality.

**Ethical considerations**

Ethical considerations pertaining to person covered principles of autonomy, informed consent, anonymity and confidentiality. However, pertaining to institution, only ethical clearance from a university and nothing is mentioned about approval by provincial gatekeepers.

**Results**

Clearly and logically articulated to facilitate repeatability.

**Discussion & implication**

Discussion relates results to available literature and is placed well within the context of existing research including what is happening in the SA health system.

**Conclusions**

This section reflects on the discussion section above.

**References**

Sources have been appropriately referenced, in text and in the list of references at the end.

**Recommendation**

Based on the above comments, it is recommended that this paper be accepted with minor corrections.
