# Supplementary material for: Navigating cancer: Insights from patient journey mapping
Source: PLoS One. 2026 Mar 25;21(3):e0327113. doi: 10.1371/journal.pone.0327113 (PMC13016344; doi:10.1371/journal.pone.0327113)
Supplement: Attachment — Submitted filename: Reviewers Table.docx [file pone.0327113.s002.docx]

**Navigating Cancer: Insights from Patient Journey Mapping**

Reviewer Response Table

| **Editor** | |
| --- | --- |
| Please ensure that your manuscript meets PLOS ONE's style requirements, including those for file naming. The PLOS ONE style templates can be found at | We have relooked at the PLOS ONE's style requirements, and made amendments to the format of the manuscript as required. |
| Thank you for stating the following financial disclosure:  [Cancer Association of South Africa (CANSA) Type B Grant].  Please state what role the funders took in the study. If the funders had no role, please state: "The funders had no role in study design, data collection and analysis, decision to publish, or preparation of the manuscript."  If this statement is not correct you must amend it as needed.  Please include this amended Role of Funder statement in your cover letter; we will change the online submission form on your behalf. | We have amended the funder statement to:  "The funders had no role in study design, data analysis or decision to publish. The funder assisted with participant recruitment but was not present during data collection. Prof Jane Harries, who is the Head of Research at CANSA, provided input on the fully drafted manuscript.” |
| Thank you for stating the following in the Acknowledgments Section of your manuscript:  [This research was supported by funding from the Cancer Association of South Africa (CANSA).]  We note that you have provided funding information that is currently declared in your Funding Statement. However, funding information should not appear in the Acknowledgments section or other areas of your manuscript. We will only publish funding information present in the Funding Statement section of the online submission form.  Please remove any funding-related text from the manuscript and let us know how you would like to update your Funding Statement. Currently, your Funding Statement reads as follows:  [Cancer Association of South Africa (CANSA) Type B Grant]  Please include your amended statements within your cover letter; we will change the online submission form on your behalf. | We have amended the acknowledgement section to say the following: “Thank you to the Cancer Association of South Africa (CANSA) and Campaigning for Cancer for their assistance with recruiting participants and providing space to conduct data collection.” |
| Thank you for stating the following in the Competing Interests section:  [SD is an academic editor for PLOSOne. All other authors have no competing interests to declare.].  Please confirm that this does not alter your adherence to all PLOS ONE policies on sharing data and materials, by including the following statement: "This does not alter our adherence to PLOS ONE policies on sharing data and materials.” (as detailed online in our guide for authors http://journals.plos.org/plosone/s/competing-interests). If there are restrictions on sharing of data and/or materials, please state these. Please note that we cannot proceed with consideration of your article until this information has been declared.  Please include your updated Competing Interests statement in your cover letter; we will change the online submission form on your behalf. | I have confirmed that this does not alter my adherence to all PLOS ONE policies on sharing data and materials, by including the following statement "This does not alter our adherence to PLOS ONE policies on sharing data and materials” in the manuscript and cover letter. However, data cannot be shared publicly as it would breach compliance with the protocol approved by your research ethics board, as it would compromise patient privacy. |
| 5. In the online submission form, you indicated that [Data cannot be shared publicly because of issues of confidentiality. Data can be made available at reasonable request.].  All PLOS journals now require all data underlying the findings described in their manuscript to be freely available to other researchers, either 1. In a public repository, 2. Within the manuscript itself, or 3. Uploaded as supplementary information.  This policy applies to all data except where public deposition would breach compliance with the protocol approved by your research ethics board. If your data cannot be made publicly available for ethical or legal reasons (e.g., public availability would compromise patient privacy), please explain your reasons on resubmission and your exemption request will be escalated for approval. | We have amended the data sharing statement to:  “Data cannot be shared publicly because public deposition would breach compliance with the protocol approved by your research ethics board, as it would compromise patient privacy. The data contains detailed accounts of patient journeys through the healthcare system, which may make them identifiable.” |
| 6. Please amend the manuscript submission data (via Edit Submission) to include author Sarah Day. | Sarah Day and Sarah Kathrine Day are the same author. We have amended the submission data to acknowledge this. |
| 7. Please amend your authorship list in your manuscript file to include author Sarah Kathrine Day. | Sarah Day and Sarah Kathrine Day are the same author. We have amended the manuscript file to acknowledge this. |
| 8. Your ethics statement should only appear in the Methods section of your manuscript. If your ethics statement is written in any section besides the Methods, please move it to the Methods section and delete it from any other section. Please ensure that your ethics statement is included in your manuscript, as the ethics statement entered into the online submission form will not be published alongside your manuscript. | Ethics only appears in the Methods section of the manuscript. |
| 9. We note that Figure 1 in your submission contains copyrighted images. All PLOS content is published under the Creative Commons Attribution License (CC BY 4.0), which means that the manuscript, images, and Supporting Information files will be freely available online, and any third party is permitted to access, download, copy, distribute, and use these materials in any way, even commercially, with proper attribution. For more information, see our copyright guidelines: <http://journals.plos.org/plosone/s/licenses-and-copyright>  We require you to either (1) present written permission from the copyright holder to publish these figures specifically under the CC BY 4.0 license, or (2) remove the figures from your submission:  1. You may seek permission from the original copyright holder of Figure 1 to publish the content specifically under the CC BY 4.0 license.  We recommend that you contact the original copyright holder with the Content Permission Form (http://journals.plos.org/plosone/s/file?id=7c09/content-permission-form.pdf) and the following text:  “I request permission for the open-access journal PLOS ONE to publish XXX under the Creative Commons Attribution License (CCAL) CC BY 4.0 (http://creativecommons.org/licenses/by/4.0/). Please be aware that this license allows unrestricted use and distribution, even commercially, by third parties. Please reply and provide explicit written permission to publish XXX under a CC BY license and complete the attached form.”  Please upload the completed Content Permission Form or other proof of granted permissions as an "Other" file with your submission.  In the figure caption of the copyrighted figure, please include the following text: “Reprinted from [ref] under a CC BY license, with permission from [name of publisher], original copyright [original copyright year].”  2. If you are unable to obtain permission from the original copyright holder to publish these figures under the CC BY 4.0 license or if the copyright holder’s requirements are incompatible with the CC BY 4.0 license, please either i) remove the figure or ii) supply a replacement figure that complies with the CC BY 4.0 license. Please check copyright information on all replacement figures and update the figure caption with source information. If applicable, please specify in the figure caption text when a figure is similar but not identical to the original image and is therefore for illustrative purposes only. | Figure 1 was created by the lead author of this manuscript, Sarah Day, specifically for this manuscript. There are no copyrighted images. |
| 10. We note that your paper includes detailed descriptions of individual patients/participants. As per the PLOS ONE policy (http://journals.plos.org/plosone/s/submission-guidelines#loc-human-subjects-research) on papers that include identifying, or potentially identifying, information, the individual(s) or parent(s)/guardian(s) must be informed of the terms of the PLOS open-access (CC-BY) license and provide specific permission for publication of these details under the terms of this license. Please download the Consent Form for Publication in a PLOS Journal (http://journals.plos.org/plosone/s/file?id=8ce6/plos-consent-form-english.pdf). The signed consent form should not be submitted with the manuscript, but should be securely filed in the individual's case notes. Please amend the methods section and ethics statement of the manuscript to explicitly state that the patient/participant has provided consent for publication: “The individual in this manuscript has given written informed consent (as outlined in PLOS consent form) to publish these case details. If the reviewer comments include a recommendation to cite specific previously published works, please review and evaluate these publications to determine whether they are relevant and should be cited. There is no requirement to cite these works unless the editor has indicated otherwise.  Please review your reference list to ensure that it is complete and correct. If you have cited papers that have been retracted, please include the rationale for doing so in the manuscript text, or remove these references and replace them with relevant current references. Any changes to the reference list should be mentioned in the rebuttal letter that accompanies your revised manuscript. If you need to cite a retracted article, indicate the article’s retracted status in the References list and also include a citation and full reference for the retraction notice. | As some participants have passed away since participating in the study and cannot sign the PLOS One Consent Form for Publication, we have elected to remove detailed descriptions of all participants. We have modified Table 1. Participants chose their own pseudonyms as part of the data collection process, and we have signed informed consent forms with their preferred pseudonym written. |
| The PLOS Data policy requires authors to make all data underlying the findings described in their manuscript fully available without restriction, with rare exception (please refer to the Data Availability Statement in the manuscript PDF file). The data should be provided as part of the manuscript or its supporting information, or deposited to a public repository. For example, in addition to summary statistics, the data points behind means, medians and variance measures should be available. If there are restrictions on publicly sharing data—e.g. participant privacy or use of data from a third party—those must be specified. | Data cannot be shared publicly because public deposition would breach compliance with the protocol approved by your research ethics board, because it would compromise patient privacy. The data contains in detail accounts of patient journeys through the healthcare system, which will make them identifiable. |
| **Reviewer 1** | |
| Thank you for the opportunity to review this interesting and important topic. Research shows that cancer is the world’s biggest killer, with 10 million deaths per year due to the disease.  Abstract  The abstract does provide a background and introduction of the study.  Main study  Introduction  The presence of important and current background information on the study topic is noted and appreciated. | Thank you for your generous comments and your invaluable feedback, that has certainly strengthened this manuscript. |
| Methods  Mapping patient journey is an interesting and unique research design appropriate to understand patients’ cancer journeys across the continuum, highlight disparities, and gaps in access and care. Sampling, data collection instruments, data collection and analysis methods have been clearly described and applied well to yield the reported results. However, researchers are not explicit as to how participant recruitment and data collection was done in the three different provinces.  How it is written can confuse the reader, e. g, “map of the municipality where data collection took place” can make one think data collection took place in one province and one municipality. | Thank you for pointing out the need for additional information on how we recruited and this potential area for confusion. We have added more details on the recruitment in each province:  “We contacted relevant stakeholders at each organisation and requested the number of participants required, participant eligibility criteria and the proposed dates and venues for data collection. These stakeholders then invited potential participants to join the study.”  “Data collection took place in a rented room at a public library in the Western Cape, and in the boardrooms of our NGO partners in Gauteng and KwaZulu-Natal.”  To reduce confusion, we amended the below sentence to the following:  “Originally, we planned for participants to co-create a map together on an A0 map of the province where the particular focus group discussion took place.” |
| Ethical considerations  Ethical considerations pertaining to person covered principles of autonomy, informed consent, anonymity and confidentiality. However, pertaining to institution, only ethical clearance from a university and nothing is mentioned about approval by provincial gatekeepers. | No additional provincial approvals were needed. We recruited through NGO partners and not through health facilities. Data collection also did not take place at health facilities. For clarity, we have added: “Data collection took place in a rented room at a public library in the Western Cape, and in the boardrooms of our NGO partners in Gauteng and KwaZulu-Natal.” |
| Results  Clearly and logically articulated to facilitate repeatability.  Discussion & implication  Discussion relates results to available literature and is placed well within the context of existing research including what is happening in the SA health system.  Conclusions  This section reflects on the discussion section above.  References  Sources have been appropriately referenced, in text and in the list of references at the end. | Thank you for your generous comments. |
| **Reviewer 2** | |
| Thank you for the opportunity to review this beautifully written paper. The authors have provided a very clear description of the cancer situation in South Africa, the patient journey mapping process they chose, underpinned by a strengths based narrative.  The very clear description of the process of individual mapping, collective mapping, producing a collective visual journey map, coding and theme development using interpretive phenomenological analysis nVivo, informed by theoretical underpinnings of intersectionality and health equity makes this paper a very important paper in the emerging and growing field of journey mapping, and also in cancer care.  Introduction provides a clear picture of the situation relating to breast and cervical cancer in South Africa, and the use of journey mapping to explore the cancer journeys of people internationally.  Research design is well described and the SA health system context provided. Is very helpful for an international readership. The theoretical underpinnings of intersectionality and health equity give the study and paper depth, and as discussed moves from a uni-dimensional view of their lived experiences to a multidimensional nuanced understanding of their cancer journey.  The description of the pilot study and participant preferences is very helpful for other studies. The three step process undertaken in focus groups is clearly and clearly explained (it sounds very effective).  The analysis process and identification of codes and themes using interpretive phenomenological analysis and NVivo is well described.  Findings – very comprehensive. | Thank you for your generous comments and your invaluable feedback, that has certainly strengthened this manuscript. |
| Line 258 – re pain management being insufficient – was this for both public and private patients? | Thank you for pointing out the lack of specificity. We have specified: “…, predominantly in the public sector due to more challenging diagnostic pathways,…” |
| Discussion  The authors could perhaps include more emphasis on specific recommendations for the women, or themselves for health system improvement in the discussion. (optional due to word length) | Thank you for your recommendation – we agree that it will make a stronger discussion. We have included the following recommendations directly from participants:  “Participants in both the public and private sector had trouble in navigating life after diagnosis and treatment. Specifically, once treatment is completed, participants reported a lack of psychosocial support in the public sector while for those on medical aid schemes they still need to pay out of pocket for rehabilitation and psychosocial health services (e.g. occupational therapy, physical therapy, psychologists and so forth) due to depleted funds. NGOs fill an important gap related to health information and psychosocial support throughout the care continuum. Participants recommended additional health information post-treatment on what to expect, long-term side effects, recurrence and required check-ups. Participants with breast cancer in the public sector reported that the compression wear for lymphoedema does not match their skin tones and recommended diverse options.”  “The study participants recommended that additional community outreach is needed to improve the public’s knowledge of cancer risk factors and symptoms in order to provide tools for patients to advocate for better care in health facilities and address stigma and misinformation.”  “In the feedback sessions, the participants recommended that, “Doctors must manage us as patients, we are not numbers”. Elaborating further, they stated that healthcare workers need to manage patients holistically and use communication strategies that are empathetic, respectful, transparent and centre consent. Within teaching facilities, participants recommended that healthcare workers talk directly to them rather than amongst themselves. Health information to prepare newly diagnosed patients on how to manage living with cancer should start at the first consultation, and not after treatment consultations. Additionally, they recommend that healthcare workers need to “hear patient voices” through translation of research findings. One example that was given was turning the findings from this study into a graphic novel which can be used by healthcare workers to prepare newly diagnosed patients on how to manage living with cancer.” |
| Conclusion – I encourage the authors to lengthen the conclusion by a few sentences to more fully emphasise the key points they would like the reader to take away. This might include the voices, the patient journey mapping method incorporating both individual and collective mapping and emerging themes, underpinned by theoretical underpinnings of intersectionality and health equity to identify both strengths and limitations of health care interactions, services and systems. | We have lengthened the conclusion to emphasise key points. The conclusion now reads:  “Grounded in intersectionality and health equity frameworks, this study used patient journey mapping - combining individual and collective mapping - to explore how women with breast and cervical cancer navigate and negotiate care in complex and unequal healthcare systems in three provinces in South Africa. Going beyond tracing clinical pathways, this study examined social, economic and affective contours of cancer care and how these factors produce differentiated access, healthcare experiences and lived realities. We traced how patients navigate and negotiate care across public and private sectors, exposing both systemic barriers and practices of humanising care within structural constraints. Within these systems, patients are not passive recipients of care: their narratives demonstrate agency, self-advocacy, and acts of resistance that have the potential to reconfigure interactions with healthcare workers and services. Patient journey mapping is a valuable approach for identifying inequities and actionable opportunities for system redesign. By translating patient experiences into visual and collective insights, it can inform more responsive, equitable and compassionate models of cancer care. Strengthening communication, continuity and support across the care continuum remains central to building a health system that truly reflects and responds to patient realities.” |
